# Comparative assessment of antitumor effects between doxorubicin and mitochondria-targeted doxorubicin in combination with radiotherapy

**DOI:** 10.32604/or.2025.058997

**Published:** 2025-05-29

**Authors:** JIANMIAO YANG, XIAOYAN SUN, TIANTIAN WANG, HAIQING ZHONG, MIN HAN, WUPING SHUAI, DONGHANG XU

**Affiliations:** 1 Department of Pharmacy, The Second Affifiliated Hospital, College of Medicine, Zhejiang University, Hangzhou, 310009, China; 2 Taizhou Hospital of Zhejiang Province, Zhejiang University, Taizhou, 317099, China; 3 Institute of Pharmaceutics, College of Pharmaceutical Sciences, Zhejiang University, Hangzhou, 310058, China; 4 Department of Clinical Pharmacy, The First Affiliated Hospital, Zhejiang University School of Medicine, Hangzhou, 310009, China; 5 Zhejiang Provincial Key Laboratory for Drug Evaluation and Clinical Research, Hangzhou, 310003, China; 6 Zhejiang Provincial Key Laboratory of Traditional Chinese Medicine for Clinical Evaluation and Translational Research, Hangzhou, 310003, China

**Keywords:** Mitochondrial-targeting, Triphenylphosphine-doxorubicin (TPP-DOX), Radiotherapy, Antitumor

## Abstract

**Objectives:**

Triphenylphosphine (TPP) and Doxorubicin (DOX) were conjugated to obtain Triphenylphosphine-Doxorubicin (TPP-DOX), which was applied in tumor cells for enhancement of DOX in mitochondria targeting. The study focused on investigating the anti-tumor effect of TPP-DOX in combination with radiotherapy throughout *in vitro* and *in vivo* studies.

**Methods:**

TPP-DOX was synthesized using the carbodiimide method. *In vitro* experiments were conducted with 4T1 cells (mouse breast cancer cell line) to assess apoptosis induction, mitochondrial targeting, reactive oxygen species (ROS) production, and mitochondrial membrane potential. The research evaluates the effects of TPP-DOX, DOX, and their combinations with radiotherapy. A nude mouse tumor heterograft model was established to investigate the synergistic effect of TPP-DOX and radiotherapy.

**Results:**

TPP-DOX was successfully synthesized and scrupulously verified. *In vitro* experiments showed that compared to DOX, TPP-DOX exhibited enhanced tumor cytotoxicity, improved cellular uptake in 4T1 cells, and increased apoptosis induction. Combined with radiotherapy, TPP-DOX promoted mitochondrial ROS production, reduced mitochondrial membrane potential, and amplified its anti-tumor effect. *In vivo* experiment confirmed that TPP-DOX combined with radiotherapy exhibited superior anti-tumor activity, promoted tumor tissue apoptosis, inhibited tumor angiogenesis, and showed a favorable *in vivo* safety profile.

**Conclusion:**

The study confirmed that when combined with radiotherapy, TPP-DOX promoted tumor cell apoptosis, and effectively enhanced the anti-tumor effect. In sensitive cells, TPP-DOX demonstrates comparable efficacy to DOX when combined with radiotherapy. TPP-DOX holds significant potential for a broader spectrum of applications and emerges as a valuable candidate for clinical application. These findings provide a promising and efficient therapeutic strategy for tumor treatment with improved efficacy and safety.

## Introduction

The incidence of cancer continues to rise steadily. According to statistics by 2022, there were approximately 20 million new cancer cases and 9.7 million deaths globally [1,2]. Among these cases, breast cancer accounts for 11.6%, surpassing lung cancer as the most prevalent malignant tumor and the leading cause of female mortality [3–5] Radiotherapy, a treatment method utilizing high-energy rays to locally target organisms, has been established as one of the standard treatments for locally advanced breast cancer [6]. Chemotherapy, on the other hand, selectively demonstrates cancer cell proliferation and induces cancer cell apoptosis, making it one of the primary treatment modalities for breast cancer. Anthracyclines, such as doxorubicin (DOX) [7], play a vital role in first-line chemotherapy regimens for breast cancer [8,9] and are widely utilized in the clinical treatment of various other cancer [10,11], like bladder cancer [12,13], lung cancer [14], stomach cancer [15,16], leukemia [17,18] ovarian cancer [19,20]. Recent studies also increasingly highlight the use of combinational strategies in breast cancer therapy, such as the application of nanostructures for chemo-radiotherapy and the combination of antisense therapy with radiotherapy [21].

However, current anti-tumor therapy faces significant challenges, like radiation resistance [22], severe damage to normal tissues [23]. Therefore, enhancing the effectiveness of localized treatments while minimizing damage to healthy tissues remains to be a challenge in radiotherapy. Additionally, individualized low-dose radiotherapy often fails to yield satisfactory results due to the limited radiation tolerance of the human body [24,25]. Furthermore, the short lifetime and limited diffusion distance of generated reactive oxygen species (ROS) result in low anti-tumor efficiency and suboptimal therapeutic effects [26,27]. Tumor tissues are heterogeneous and exhibit characteristics such as chemotherapy resistance, radiotherapy resistance, and hypoxia resistance [28–30]. As sensitive tumor cells eliminates, some cells are induced to develop drug resistance, resulting in chemotherapy failure or tumor recurrence [31,32]. Merely augmenting the dose of chemotherapy drugs to overcome drug resistance may lead to severe adverse reactions in patients [33].

By promoting organelle-specific drug distribution within cells, the anti-tumor effects of drugs can be further enhanced [34,35]. Mitochondria, with their high membrane potential and significant involvement in cell growth, metabolism, apoptosis, drug resistance, and metastasis, are in particular relevance with tumor cells [36–39]. Mitochondria also generate up to 90% of ROS in cells [40], making them crucial in apoptosis, a major cancer therapy mechanism, and sensitization to radiotherapy [41,42]. Dysfunctional mitochondria contribute to imbalances in fission and fusion, and increased expression of related proteins is associated with tumor cell migration [43,44].

DOX, an anthracycline antineoplastic drug widely employed in clinical practice, exerts its effects primarily on DNA [45]. Mitochondria possess a phospholipid bilayer and carry a negative charge (−140 mV) [46]. Considering their lipophilic cationic structure, molecules such as triphenylphosphine (TPP) [47], containing three benzene rings, can be employed to specifically target mitochondria [48,49]. Therefore, we developed a novel approach to synthesize triphenylphosphine-doxorubicin (TPP-DOX) with properties of mitochondrial targeting based on triphenylphosphine to achieve radiotherapy sensitization. Deliver DOX to mitochondria and improve mitochondrial drug accumulation, furtherly combined with radiotherapy to enhance its antitumor activity [49]. Upon X-rays irradiation, it can generate ROS in mitochondria, leading to mitochondrial collapse and irreversible apoptosis, which may provide new insights into radiotherapeutic sensitization in future clinical cancer treatment [35]. Overall, these research findings provide valuable insights into the application of TPP-DOX for mitochondria targeting in tumor treatment, offering significant implications for future endeavors in this field.

## Materials and Methods

### Materials

Doxorubicin hydrochloride (DOX·HCl) (Dalian Meilun Biotechnology Co., Ltd., MB1087, Dalian, China); (3-carboxypropyl) triphenylphosphine bromide (TPP) (Shanghai Aladdin Biochemical Technology Co., Ltd., C119836, Shanghai, China); N-Hydroxysuccinimide (NHS) (Shanghai Macklin Biochemical Technology Co., Ltd., N811124, Shanghai, China); N,N′-dicyclohexylcarbodiimide (DCC) (Shanghai Macklin Biochemical Technology Co., Ltd., N806920); Hydrochloric acid (HCl) (Sinopharm Chemical Reagent Co., Ltd., 10011018, Beijing, China); N,N-Dimethylformamide(DMF) (Saen Chemical Technology Co., Ltd., C0425510223, Shanghai, China); Triethylamine (TEA) (Sinopharm Chemical Reagent Co., Ltd., TMLT18954LMG1); Anhydrous ether (Sinopharm Chemical Reagent Co., Ltd., 10009328); Trichloromethane (Sinopharm Chemical Reagent Co., Ltd., 10006818). Gibco fetal bovine serum (Thermo Fisher Scientific, A5670701, Shanghai, China); RPMI-1640 complete medium (Zhejiang Senrui Biotechnology Co., Ltd., CR-31800, Huzhou, China); 0.25% trypsin & 0.02% EDTA (Zhejiang Senrui Biotechnology Co., Ltd., CR-25200); 4% Paraformaldehyde (Beijing Suolaibao Technology Co., Ltd., P1110, Beijing, China); Penicillin-streptomycin double antibody solution (Tianjin Haoyang Biological Products Technology Co., Ltd., TBD20180091, Tianjin, China); CCK-8 cytotoxicity detection kit (Shanghai Beyotime Biotechnology Co., Ltd., C0037, Shanghai, China); Apoptosis detection Reagent kit (Shanghai Yisheng Biotechnology Co., Ltd., 40311ES20, Shanghai, China); Active oxygen detection kit (Shanghai Beyotime Biotechnology Co., Ltd., S0033S); Hoechst 33342 staining solution (Shanghai Beyotime Biotechnology Co., Ltd., C1022); Hanks buffer (Shanghai Macklin Biochemical Technology Co., Ltd., H917811); Calcein AM/PI double staining kit (Elabscience Biotechnology Co., Ltd, E-CK-A354, Wuhan, China); Mitochondrial membrane potential detection kit (JC-1) (Shanghai Beyotime Biotechnology Co., Ltd., C1071M); Mitochondrial fluorescent probe (Mito-Tracker) (Shanghai Beyotime Biotechnology Co., Ltd., C1032); Caspase kit (Shanghai Beyotime Biotechnology Co., Ltd., C1168M); Ki67 Cell Proliferation Assay Kit (Shanghai Beyotime Biotechnology Co., Ltd., C2312S); Cytochrome C antibody at dilution of 1:200 (Shanghai Beyotime Biotechnology Co., Ltd., AC909); CD31 Rabbit Polyclonal Antibody at dilution of 1:100 (Shanghai Beyotime Biotechnology Co., Ltd., AF6408); Immunol Staining Blocking Buffer (Shanghai Beyotime Biotechnology Co., Ltd., P0102); p63 Rabbit Monoclonal Antibody (Shanghai Beyotime Biotechnology Co., Ltd., AG8597); TUNEL Apoptosis Detection Kit (YEASEN biotechnology (Shanghai) Co., Ltd., 40306ES60, Shanghai, China); Hematoxylin-eosin (H&E) staining (Shanghai Beyotime Biotechnology Co., Ltd., C0105S); Mitochondrial DNA detection kit (Wuhan Servicebio Technology CO., LTD, GM2019, Wuhan, China); ATP Assay Kit (MedChemExpress, HY-K0314, Shanghai, China); MCF-10A cell-specific medium (Wuhan Pricella Biotechnology Co., Ltd., CM-0525, Wuhan, China).

### Methods

#### Synthesis of TPP-DOX

**Figure 1:**
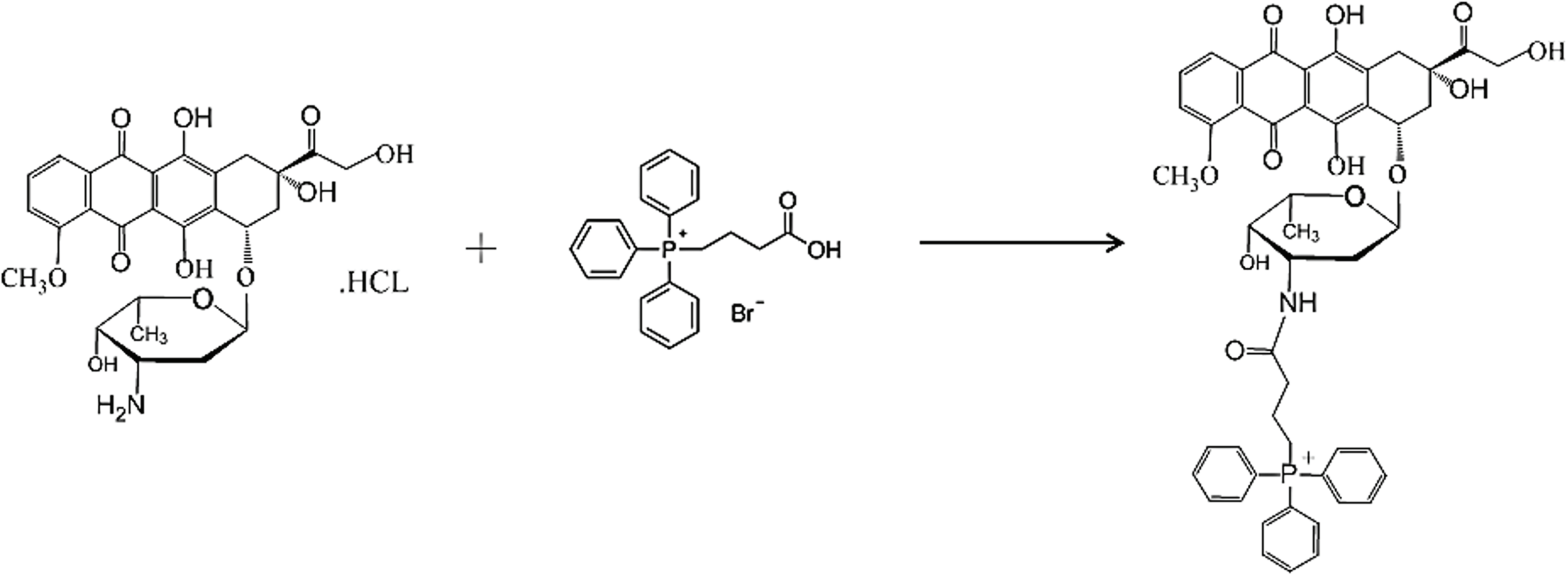
The synthesis process of TPP-DOX.

The TPP-DOX was synthesized by using active TPP and alkaline DOX (Fig. 1). To synthesize TPP-DOX, TPP (45 mg) was dissolved in DMF (10 mL) for 10 min. DCC (20 mg) and NHS (14 mg) were then added to the above solution. The reaction mixture was stirred at room temperature for 3 h and centrifuged at 4000 r·min^−1^ with Benchtop centrifuge (LT53, cence) for 10 min to collect the supernatant. In parallel, DOX·HCl (60 mg) was dissolved in DMF (15 mL), and triethylamine was added to alkalize the DOX·HCl. The active TPP was added dropwise to the alkaline DOX and stirred overnight at room temperature, protected from light [50,51].

The resulting reaction product was mixed with 400 mL of anhydrous ether and allowed to stand for 24 h. The upper ether layer was carefully discarded, and the red precipitate was collected. The precipitate was then subjected to chloroform ultrasonic extraction in a 50 mL eggplant-shaped bottle. The chloroform extract was collected and the solvent was removed using a rotary evaporator (RE52AAA, Shanghai Jiapeng Technology Co., Ltd., Shanghai, China). The resulting precipitate was repeatedly extracted until the chloroform layer no longer showed obvious red color. The collected extract was further purified using silica gel to prepare thin layer plate chromatography.

#### Quantification analysis method

For quantification analysis, TPP-DOX was dissolved in a suitable deuterated solvent and analyzed using ^1^H nuclear magnetic resonance (^1^H NMR, Agilent 6460, Agilent Technologies, Palo Alto, AK, USA). Additionally, TPP-DOX was subjected to mass spectrometry analysis using Liquid Chromatograph-Mass Spectrometer (LC-MS, AB SCIEX QTRAP 5500, AB SCIEX, USA) to determine its molecular weight and infer its structure. Furthermore, a portion of TPP-DOX powder was taken and ground with potassium bromide to prepare a sample for Fourier transform infrared (FT-IR) analysis to study the structural properties of the product.

#### Cell culture

4T1 cells (mouse breast cancer cell line, National collection of authenticated cell culture, Shanghai, China, Mycoplasma negative) and HUVEC (Immortal human umbilical cord vein endothelial cells, HUVEC+SV40, National collection of authenticated cell culture, Shanghai, China, Mycoplasma negative) were cultured in RPMI-1640 complete medium supplemented with 1% penicillin-streptomycin and 10% Gibco fetal bovine serum (FBS) at 37°C in a humidified 5% CO_2_ incubator (HF151UV, Heal Force, Hong Kong, China). MCF-10A (human mammary epithelial cell, CRL-10317, Wuhan Pricella Biotechnology Co., Ltd., Wuhan, China, Mycoplasma negative) were cultured in MCF-10A cell-specific medium at 37°C in a humidified 5% CO_2_ incubator.

#### Cytotoxicity assay in vitro

8 × 10^3^ 4T1 cells were seeded in each well of a 96-well plate and cultured for 24 h. Two groups, one with irradiation (5 Gy) and one without irradiation (0 Gy), were set up with 6 replicates each. The culture medium was aspirated, and different concentrations of DOX and TPP-DOX drug-containing culture solutions (0.125, 0.25, 0.50, 1.0 μg/mL) were added to each well. After incubating for 6 h, the drug solution was removed and washed with PBS. The irradiation group was then exposed to X-ray irradiation at a dose of 5 Gy [52–54] and further incubated for 24 h. The cell viability was measured using a microplate reader (Bio-Tek ELX800, BioTek, VT, USA) at an absorbance of 450 nm after adding a CCK-8 detection reagent to each well.

8 × 10^3^ HUVEC or MCF-10A cells were seeded in each well of a 96-well plate and cultured for 48 h. Two groups, one with irradiation (5 Gy) and one without irradiation (0 Gy), were set up with 6 replicates each. The culture medium was aspirated, and different concentrations of DOX and TPP-DOX drug-containing culture solutions (0.125, 0.25, 0.50, 1.0 μg/mL) were added to each well. After incubating for 6 h, the drug solution was removed and washed with PBS. The irradiation group was then exposed to X-ray irradiation at a dose of 5 Gy and further incubated for 24 h. The cell viability was measured using a microplate reader (Bio-Tek ELX800, BioTek) at an absorbance of 450 nm after adding a CCK-8 detection reagent to each well.

Furthermore, cells were cultured in a glass-bottomed 35 mm culture dish for 24 h. Following the removal of the medium, cells were treated with DOX or TPP-DOX (at concentrations of 0.125 and 1 μg/mL) and incubated for 6 h. Subsequently, the culture medium was removed, and the cells were irradiated with 5 Gy X-rays, followed by continued incubation for an additional 24 h. Staining procedures were performed as Calcein AM/PI double staining kit protocol, and cellular observations were conducted using a Confocal Microscope (TCS SP8, Leica Biosystems, Wetzlar, Germany). Semi-quantitative analysis was performed using ImageJ (IJ154-win-java8, National Institutes of Health, Bethesda, MD, USA).

#### Cell uptake studies

Cell uptake studies were conducted on 4T1 cells. The cells were seeded in a 96-well plate at a concentration of 8 × 10^3^ cells per well and cultured for 12 h. Culture solutions containing DOX and TPP-DOX were added to achieve a DOX concentration of 0.5 μg/mL, and incubated for 12 h. The cells were then rinsed with PBS and the drug content was detected with an excitation wavelength of 470 nm and an emission wavelength of 560 nm [55] absorbance.

#### Assessment of mitochondrial-targeting by confocal laser scanning microscopy (CLSM)

In a confocal dish, 2 × 10^5^ 4T1 cells were inoculated per well. After 24 h of culture, culture solutions containing DOX and TPP-DOX were added to achieve a final concentration of 0.5 μg/mL for DOX. Following a 6-h incubation in the incubator, the drug solution was removed through centrifugation. The radiotherapy group underwent a 5 Gy radiotherapy treatment. Subsequently, the cells were stained with Hoechst 33342 and Mito-Tracker and immersed in Hanks buffer containing 10% FBS. Finally, the distribution of the drugs within the cells was examined using a confocal microscope (TCS SP8, Leica Biosystems, Wetzlar, Germany).

#### Apoptosis research

Annexin V is capable of selectively binding to phosphatidylserine, effectively blocking coagulation and pro-inflammatory response upon binding. Propidium iodide can be used to stain cells with compromised membrane integrity, specifically necrotic cells in the late stage of apoptosis, resulting in red fluorescence. Necrotic cells exhibit green fluorescence due to the loss of membrane integrity, allowing Annexin V-FITC to enter the cytoplasm and bind to phosphatidylserine on the inner side of the cell membrane.

A six-well plate was utilized, and 2 × 10^5^ 4T1 single cells were inoculated per well. After 24 h of culture, DOX and TPP-DOX-containing culture solutions (with PBS as the control) were added, achieving a final concentration of 0.5 μg·mL^−1^ for DOX. Following a 6-h incubation in the incubator, the drug solution was removed by centrifugation, and fresh culture solution was added for 12 h. The cell culture medium was then collected, washed with PBS, and digested with an appropriate amount of trypsin. The adherent cells were gently pipetted at room temperature, and the trypsin cell digestion solution was discarded. The collected cell culture medium was added, pipetted down, transferred to a centrifuge tube, and centrifuged at 1000 g for 5 min. The cells were collected, resuspended in PBS, and counted. Subsequently, 1 × 10^5^ resuspended cells were centrifuged at 1000 g for 5 min, the supernatant was discarded, and the cells were resuspended in 195 μL Annexin V-FITC conjugate solution. Finally, 5 μL Annexin V-FITC and 10 μL propidium iodide staining solution were added, mixed thoroughly, and incubated for 10 min at room temperature in the dark. Flow cytometry analysis was then conducted.

Additionally, for cell culture in the six-well plate, treat with 0.5 μg/mL DOX for 6 h. Digest adherent cells with trypsin, collect them by centrifugation, wash with PBS, and aspirate the supernatant. Prepare lysis solution (100 μL/10^6^ cells), resuspend the cells, lyse in an ice bath for 15 min, and centrifuge at 20,000 g for 15 min at 4°C. Transfer the supernatant to a pre-cooled tube in an ice bath. Follow the Caspase kit instructions to set up the reaction system, adding substrates Ac-DEVD-pNA and Ac-LEHD-pNA. Incubate for 2 h, then use a microplate reader to detect and calculate pNA concentration, comparing Caspase-3 and Caspase-9 activity levels.

#### Mitochondrial ATP production

2 × 10^5^ 4T1 cells were seeded into each well of a 6-well plate and cultured for 24 h, then treated with DOX or TPP-DOX (0.5 μg/mL) for 6 h. After irradiation with 5 Gy X-rays, cells were incubated for an additional 24 h. Following treatment, cells were collected and lysed by adding 150 μL of lysis buffer provided by the ATP detection kit. The samples were vortexed for 1 min and then centrifuged at 12,000× g at 4°C for 5 min. The supernatant was transferred to a new tube for ATP measurement. A 10 μL aliquot from each sample was mixed with 100 μL of ATP detection solution, and the mixture was thoroughly mixed. ATP levels were quantified using a fluorescent microplate reader (SpectraMax iD5, Molecular Devices, CA, USA). ATP concentration in each sample was determined by comparing the fluorescence intensity to a standard curve.

#### Mitochondrial membrane potential assay

The decrease in mitochondrial membrane potential is an early indicator of apoptosis. JC-1 is a commonly used fluorescent probe to detect mitochondrial membrane potential (∆Ψm). When the potential is low, JC-1 acts as a monomer and exhibits green fluorescence since it cannot aggregate in the mitochondrial matrix. Conversely, when the potential is high, JC-1 can aggregate in the mitochondrial matrix, forming polymers and producing red fluorescence.

2 × 10^5^ 4T1 single cells were inoculated per well in a confocal dish. After 24 h of culture, culture solutions containing DOX and TPP-DOX were added to achieve a final concentration of 0.5 μg/mL for DOX. Following a 6-h incubation in the incubator, the drug solution was removed by centrifugation. The radiotherapy group received a 5 Gy radiotherapy treatment and was subsequently stained with JC-1 dye. The positive control group’s cells were treated with Carbonyl cyanide 3-chlorophenylhydrazone (CCCP), provided in the kit, for 20 min. The changes in mitochondrial membrane potential were observed using a confocal microscope (TCS SP8, Leica Biosystems).

#### Mitochondrial DNA (mtDNA) quantity and quality

2 × 10^5^ 4T1 cells were seeded into each well of a 6-well plate and cultured for 24 h, then treated with DOX or TPP-DOX (0.5 μg/mL) for 6 h. After irradiation with 5 Gy X-rays, cells were incubated for an additional 24 h. The procedure was carried out following the instructions provided with the mtDNA detection kit, and the analysis was performed using a fluorescent quantitative PCR instrument (CFX Connect, Bio-Rad, CA, USA).

#### Reactive oxygen species (ROS) measurements

Take a 96-well plate, inoculate 8 × 10^3^ 4T1 single cells per well, and add 100 μL of culture solution containing DOX and TPP-DOX after 24 h of culture, resulting in a final concentration of 0.5 μg/mL for DOX. After a 6 h incubation, the liquid was removed by centrifugation. The radiotherapy group underwent a 5 Gy radiotherapy treatment and was cultured overnight. Subsequently, the cells were treated with a diluted DCFH-DA probe, added to the cells, and incubated at 37°C for 20 min. The samples were then analyzed using an M5 microplate reader (MD M5, Molecular devices, CA, USA).

#### Transmission electron microscopy (TEM) of mitochondria

2 × 10^5^ 4T1 cells were seeded into each well of a 6-well plate and cultured for 24 h, then treated with DOX or TPP-DOX (0.5 μg/mL) for 6 h. After irradiation with 5 Gy X-rays, cells were incubated for an additional 24 h. The medium was discarded, and cells were fixed in 2.5% glutaraldehyde at 4°C overnight, rinsed with phosphate buffer, and post-fixed in 1% osmium tetroxide for 1–2 h. After further rinsing, cells were dehydrated in graded ethanol and acetone solutions, followed by infiltration with Spurr embedding medium. Embedded samples were polymerized at 70°C overnight, sectioned (70–90 nm), stained with lead citrate and uranyl acetate, and examined using a Hitachi HT7820 transmission electron microscope (Hitachi High-Tech Corporation, Tokyo, Japan).

#### Cell scratch

To investigate the impact of different formulations on tumor migration and metastasis, cell scratch experiments were conducted. 2 × 10^5^ 4T1 Cells in the logarithmic growth phase were seeded in a six-well plate and cultured for 24 h. Following the removal of the culture medium, a scratch was made at the bottom of each well using the tip of a pipette. Cells were then rinsed three times with PBS, and 2 mL of culture medium was added. Subsequently, DOX and TPP-DOX were individually added at a concentration of 0.5 μg/mL and incubated accordingly. The transmembrane migration of tumor cells was observed, photographed, and recorded at intervals of 0, 24, and 48 h using a microscope (Model 800, Carl Zeiss AG, Oberkochen, Germany). Semi-quantitative analysis was conducted using ImageJ (IJ154-win-java8, National Institutes of Health). The cell relative migration rate was calculated using the formula: Cell migration rate % = (Initial wound area − Final wound area)/Initial wound area × 100.

#### Body weight changes of tumor-bearing grafted nude mice

To establish a nude mouse model bearing 4T1 grafted tumors, 5-week-old BALB/c nude mice (Zhejiang Vitalriver Laboratory Animal Technology Co., Ltd., female, 18 g). The mice were housed in standard laboratory conditions with a 12-h light/dark cycle, controlled temperature (22 ± 2°C), and relative humidity (50 ± 10%). Mice had free access to food and water throughout the study. The mice were subcutaneously inoculated with 10^6^ 4T1 cells (0.1 mL).

The 4T1 cells in the logarithmic growth phase were digested and resuspended in an appropriate amount of PBS. The cell suspension was diluted to a concentration of 10^7^ cells·mL^−1^, and 0.1 mL was injected subcutaneously into the right abdomen of each nude mouse. Once the tumors reached an appropriate size, mice with uniform tumor sizes were selected and divided into 6 groups: Group A received intratumoral injection of TPP-DOX (3 mg·kg^−1^), Group B received the same dose of TPP-DOX intratumoral injection and 5 Gy radiotherapy, Group C received 5 Gy radiotherapy, Group D received intratumoral injection of DOX (1.9 mg·kg^−1^), Group E received intratumoral injection of the same dose of DOX and 5 Gy radiotherapy, and Group F served as the control group. Injections were performed on the 1st, 4th, and 7th days, while radiotherapy was administered on the 2nd, 5th, and 8th day. The total treatment cycle lasted for 9 days (Fig. 4A). The mice’s body weight was measured daily, and a body weight change curve was plotted to observe the treatment’s effect on the mice’s body weight.

#### Anti-tumor activities in tumor-bearing grafted nude mice

30 BALB/c nude mice bearing grafted tumors were selected from the previously constructed model and divided into 6 groups, with 5 mice in each group. The mice were treated according to the protocol outlined in the “Bodyweight Changes of Tumor-bearing Grafted Nude Mice Model” section. Starting from the 9th day of treatment, the tumor size of the mice was measured daily, and tumor volume was calculated using the formula V = 0.52 × L × W^2^. A tumor growth curve was plotted to observe the effect of the treatment on tumor size in the mice.

Following the euthanasia of the tumor-bearing grafted nude mice, the tumor tissues were dissected and fixed in 4% paraformaldehyde for 48 h. After paraffin sectioning, the following tests were performed according to the manufacturer’s method. Protein expression analysis was performed on the tumor tissues, including Ki67 (a proliferation marker), CYT C (leaked after tumor cell apoptosis), platelet-endothelial cell adhesion molecule CD31, tumor cell apoptosis-related indicators (such as TUNEL), and other tumor proliferation-related indicators, following the instructions provided in the respective kits. CYT C staining was performed by blocking tumor samples with blocking solution for 1 h at room temperature. After aspirating the blocking solution, samples were incubated with diluted primary antibody for 1 h at room temperature. Following washing, the Cytochrome C antibody at dilution of 1:200 was applied and incubated for 1 h at room temperature. Samples were then observed under the microscope (TCS SP8, Leica Biosystems, Wetzlar, Germany). CD31 staining was performed by blocking tumor samples with blocking solution for 1 h at room temperature. After aspirating the blocking solution, samples were incubated with diluted primary antibody for 1 h at room temperature. Following washing, the CD31 Rabbit Polyclonal Antibody was applied and incubated for 1 h at room temperature. Samples were then observed under the microscope (TCS SP8, Leica Biosystems, Wetzlar, Germany).

#### Organ toxicity evaluation in tumor-bearing grafted nude mice

30 BALB/c nude mice bearing grafted tumors were selected and divided into 6 groups, with 5 mice in each group, following the same treatment described in the “Body weight change of tumor-bearing grafted nude mice model” section. On the 9th day of treatment, the mice were sacrificed, and major organs such as the heart, liver, kidney, spleen, and lung were excised. A portion of each organ was fixed in 4% paraformaldehyde solution for 48 h to prepare tissue paraffin sections. The remaining portion of each organ was subjected to hematoxylin-eosin (H&E) staining to detect potential toxic side effects, particularly cardiotoxicity, in each group.

#### Blood routine and blood biochemistry of tumor-bearing grafted nude mice

30 BALB/c nude mice bearing grafted tumors were selected and divided into 6 groups, with 5 mice in each group, following the treatment protocol described in the “Bodyweight Changes of Tumor-bearing Grafted Nude Mice Model” section. After completing the treatment, blood samples were collected from the mice, and blood routine and blood biochemical indicators were measured using a biochemical analyzer (AU5800, BECKMAN COULTER, CA, USA).

##### Statistics

Statistical analysis was performed using GraphPad Prism 9 (GraphPad Software, CA, USA). Data were presented as means ± S.D. Statistical evaluation of differences between experimental groups was performed by one-way ANOVA followed by Tukey’s multiple comparisons test. Statistical significance was considered at least at *p* < 0.05.

## Result

### Synthesis and characterization of TPP-DOX

The hydrogen spectrum of the synthesized TPP-DOX was analyzed using the ^1^H-NMR method (Fig. S1A). The figure reveals the presence of characteristic peaks corresponding to the hydroxyl hydrogen signal (5.5 ppm) and methoxy hydrogen signal (4.0 ppm) of DOX (Fig. S1B) in the TPP-DOX product. Additionally, the characteristic peak (8.0 ppm) of triphenyl, present in TPP (Fig. S1C), was also observed, indicating the successful synthesis of TPP-DOX.

Mass spectrometry (MS) analysis of TPP-DOX (Fig. S2A) identified the molecular ion peak ([M+H]^+^) at 874.3. Comparing the mass spectrum results of DOX (Fig. S2B) and TPP (Fig. S2C), the molecular ion peaks were found at 544.2 and 349.1, respectively, further confirming the successful synthesis of TPP-DOX.

The infrared spectrum of TPP-DOX (Fig. S3) exhibited characteristic peaks at 3409 cm^−1^ (amide bond NH stretching vibration), 1724 cm^−1^ (ketone carbonyl C=O stretching vibration), 1630 cm^−1^ (amide carbonyl C=O stretching vibration), and benzene ring carbon-carbon double bond C=C characteristic peaks at 1617, 1578, 1439, and 1412 cm^−1^. A comparison of the infrared spectra of DOX and TPP-DOX revealed a more prominent benzene ring absorption in TPP-DOX at 3300–3000 cm^−1^, further confirming the combination of TPP and DOX.

### Cytotoxicity assay in vitro

**Figure 2:**
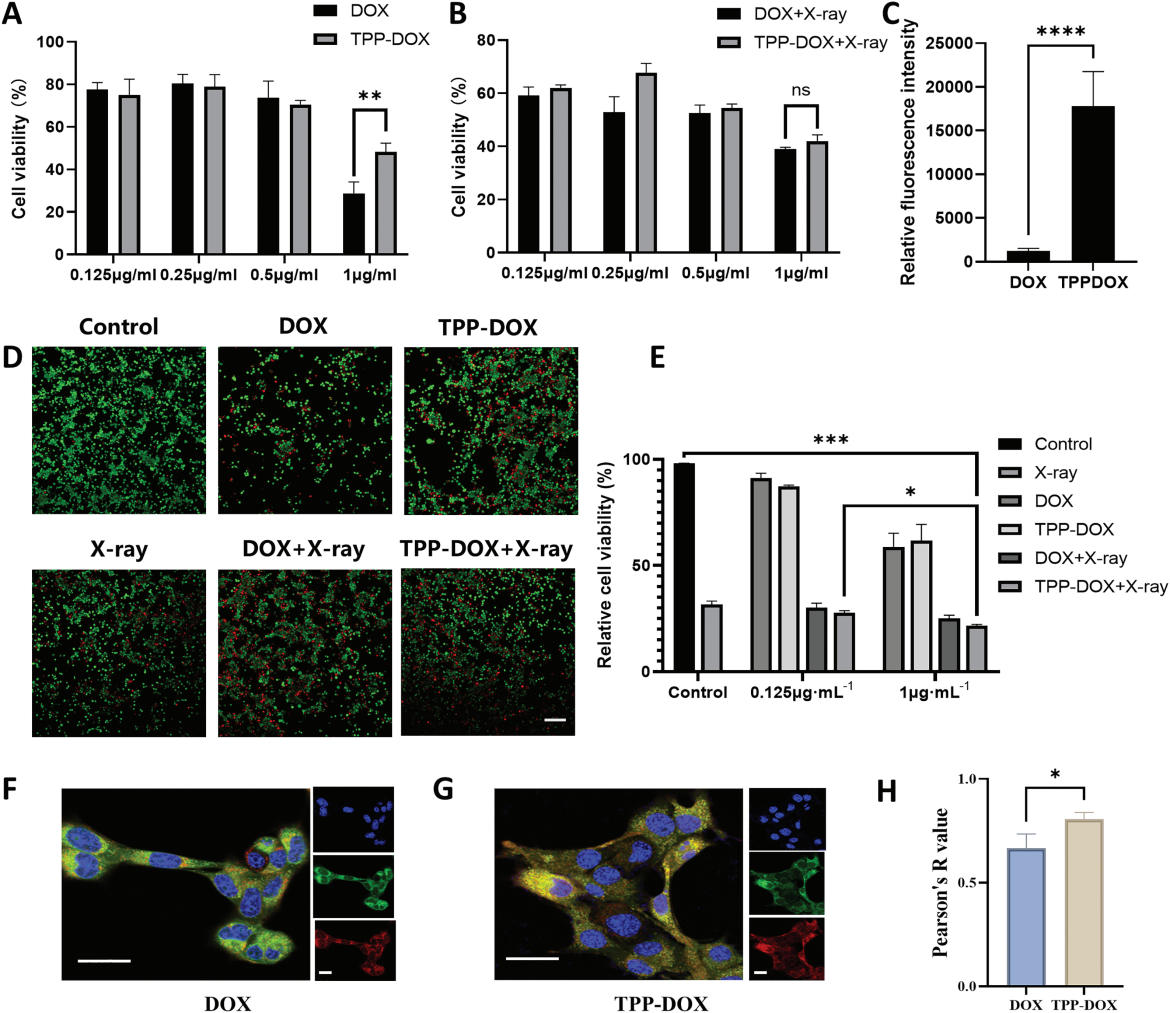
TPP-DOX targets to mitochondria with high accumulation in the cancer cells. (A) 4T1 cytotoxicity investigation of different drug treatment groups (DOX, TPP-DOX); n = 3, (**p < 0.01); (B) 4T1 cytotoxicity of different drug treatment groups (DOX, TPP-DOX) combined with radiotherapy; (n = 3, ns, *p* > 0.05,); (C) intracellular drug uptake of breast cancer 4T1 cells incubated with DOX or TPP-DOX for 12 h (n = 3, ****p < 0.0001) detected by confocal microscopy; (D) fluorescence image of 4T1 cells stained with Calcein AM/PI (bar = 200 μm); (E) fluorescence intensity qualified by ImageJ in Fig. S5 (n = 3, *p < 0.05, ***p < 0.001); (F) distribution of DOX (green) in mitochondria (red) or nucleus (blue) of 4T1 cells (bar = 40 μm); (G) distribution of TPP-DOX (green) in 4T1 cells (bar = 40 μm); (H) pearson correlation coefficients of colocalization of Mito-Tracker Red CMX ROS and drug fluorescence (n = 3, *p < 0.05).

To assess the efficacy of combined drug and radiotherapy on breast cancer 4T1 cells, the CCK-8 kit was used to evaluate the tumor cytotoxicity of different concentrations of DOX, TPP-DOX, and their respective 5 Gy doses of X-ray irradiation. At low and medium concentrations, the cytotoxicity of the free DOX treatment group (Fig. 2A) was comparable to that of the TPP-DOX treatment group, while higher concentrations of DOX exhibited stronger cytotoxicity. This observation may be attributed to the direct cytotoxic effect of DOX on the nuclear DNA of sensitive tumor cells.

After combined radiation therapy, the tumor cytotoxicity of the TPP-DOX combined with the radiation therapy group showed a significant enhancement with increasing drug concentration (Fig. 2B). In contrast, the enhancement range of the DOX combined treatment group was less apparent. Cytotoxicity results (Fig. S4A–D) show that TPP-DOX is less toxic to normal cells (HUVEC and MCF10A) than DOX, both with and without X-ray irradiation. In comparison to 4T1 tumor cells, normal cells exhibit significantly lower drug toxicity, suggesting a favorable safety profile for TPP-DOX. This may be due to the higher mitochondrial metabolic activity in tumor cells, which makes them more susceptible to TPP-DOX-induced toxicity. The results of cell viability and mortality staining reveal that while TPP-DOX exhibits a slightly reduced cytotoxicity compared to DOX (Fig. 2D), its efficacy in cell killing is markedly enhanced under the combined influence of X-ray irradiation (Fig. 2E).

### Cell uptake studies

The uptake of DOX and TPP-DOX by 4T1 cells was evaluated by confocal microscopy (Fig. 2C). The structure of TPP-DOX facilitated a higher uptake compared to DOX, possibly due to the positive charge of the lipophilic cation TPP and its interaction with negatively charged cell membranes, thereby promoting the uptake of TPP-DOX.

### Assessment of mitochondrial-targeting by confocal laser scanning microscopy (CLSM)

The intracellular drug distribution of 4T1 cells following DOX (Fig. 2F) and TPP-DOX (Fig. 2G) treatment was observed using fluorescence confocal microscopy. The fluorescence images demonstrated the clear display of drug organelle distribution in tumor cells, confirming the mitochondrial targeting of TPP-DOX. The green fluorescence in the TPP group specifically indicated the mitochondrial targeting of TPP-DOX, leading to the superposition of green and yellow signals.

Colocalization analysis using the Pearson coefficient calculation method was performed to further investigate the colocalization of TPP-DOX and mitochondria (Fig. 2H). The calculation method utilized the colocalization analysis tool in ImageJ, with the coefficient ranging from −1 to 1. The colocalization analysis demonstrated that TPP-DOX had a significantly higher coefficient closer to 1 compared to free DOX, indicating a more pronounced co-localization with mitochondria and successful mitochondrial targeting.

### Apoptosis research

**Figure 3:**
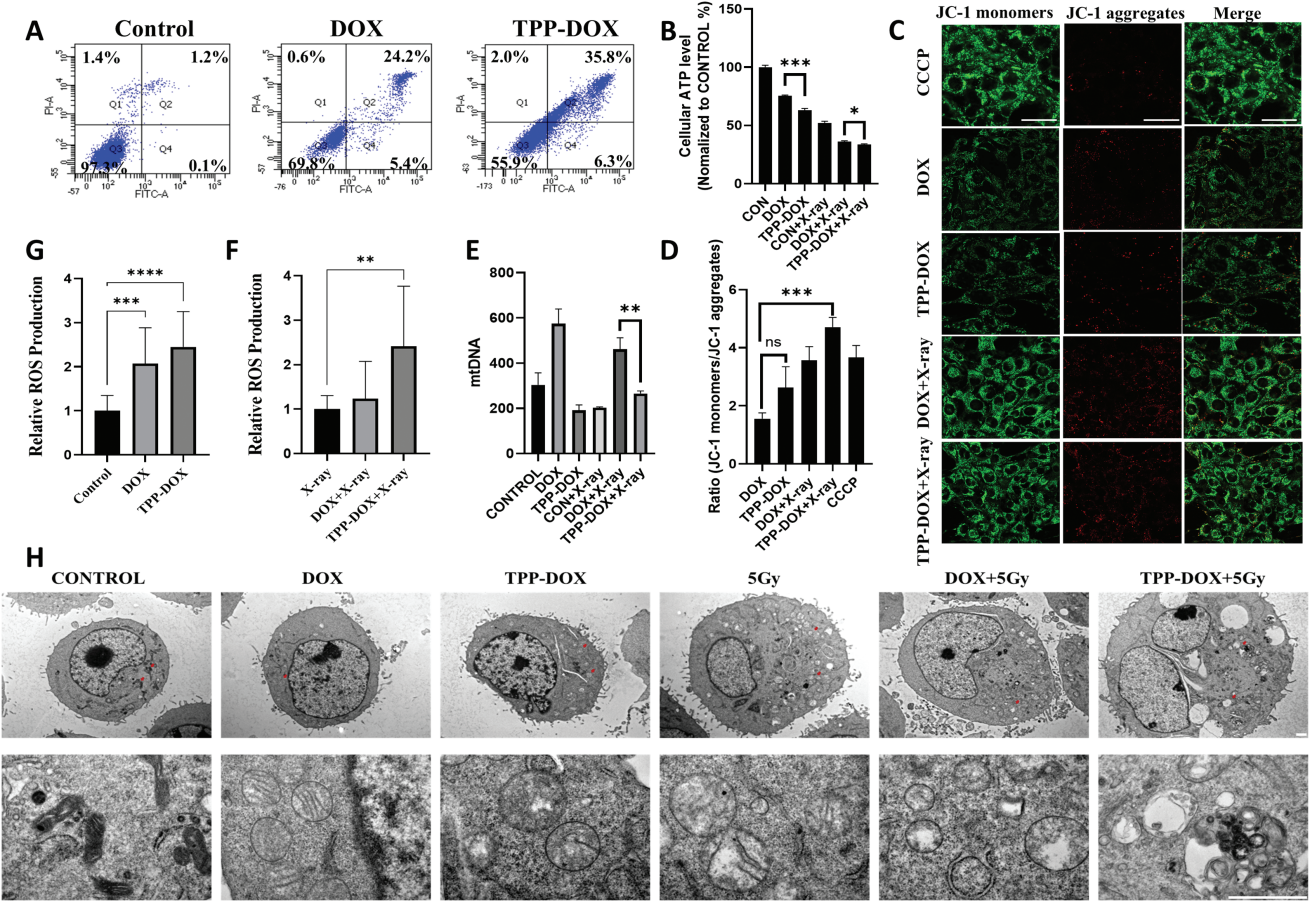
TPP-DOX induces cell apoptosis in 4T1 cells while decreasing mitochondrial membrane and increasing ROS levels. (A) Detection of apoptosis level of breast cancer 4T1 cells treated with DOX or TPP-DOX by flow cytometry; (B) ATP secretion (n = 3, *p < 0.05, ***p < 0.001); (C and D) Semi-quantitative analysis of mitochondrial membrane potential (bar = 50 μm) and green fluorescence intensity of JC-1 monomer in 4T1 cells before and after treatment with DOX or TPP-DOX combined with radiotherapy (n = 3, ns, *p* > 0.05, ***p < 0.001); (E) mtDNA quantity and quality (n = 3, **p < 0.01); (F and G) Relative production of ROS in 4T1 cells before and after DOX or TPP-DOX combined with radiotherapy (n = 18, **p < 0.01, ***p < 0.001, ****p < 0.0001); (H) transmission electron microscopy (TEM) of mitochondria (bar = 1 µm). The red arrow indicates the magnified area of the image, where the mitochondrial morphology is prominently displayed.

Flow cytometry was employed to detect cell apoptosis after treatment with TPP-DOX and DOX, followed by staining with PI and Annexin V-FITC (Fig. 3A). Both DOX and TPP-DOX induced cell apoptosis in 4T1 cells, with a higher proportion of apoptotic cells observed in the TPP-DOX treatment group. Targeting the apoptosis-related protease Caspase-3 and Caspase-9 activities of 4T1 cells, the levels of Caspase-3 and Caspase-9 in 4T1 cells treated with TPP-DOX were slightly increased (Fig. S6). This outcome suggests the potential of TPP-modified drugs in anti-tumor therapy and indicates that the mitochondrial targeting guided by TPP may be responsible for this change.

### Mitochondrial ATP production

The results demonstrated that the TPP-DOX treatment group exhibited significantly lower ATP levels compared to the DOX treatment group, regardless of whether X-rays were applied (Fig. 3B, Fig. S7). This finding suggests that TPP-DOX induces more severe mitochondrial damage. Furthermore, in the presence of X-rays, mitochondrial damage was further exacerbated, indicating that TPP-DOX effectively targets mitochondria, thereby enhancing its cytotoxic effect. This suggests that TPP-DOX, in combination with radiation, leads to more efficient tumor cell killing through its impact on mitochondrial function.

### Mitochondrial membrane potential assay

The mitochondrial membrane potential of 4T1 cells treated with different concentrations of DOX and radiation therapy was assessed using confocal microscopy (Fig. 3C). ImageJ was employed for the semi-quantitative analysis of JC-1 monomer (green fluorescence) and JC-1 aggregate (red fluorescence) intensities. The positive control group (CCCP treatment) exhibited a substantial decrease in mitochondrial membrane potential, evident in the bright green fluorescence of JC-1 monomers (Fig. 3D). Following TPP-DOX treatment, the green fluorescence intensity of JC-1 monomers was slightly stronger than that of DOX, while the fluorescence intensity was significantly enhanced in the combined radiation therapy group. Crucially, the combination of TPP-DOX with X-ray irradiation led to a substantial increase in the JC-1 monomers/JC-1 aggregates ratio. This outcome signifies a more pronounced effect in reducing mitochondrial membrane potential. This finding suggests that TPP-DOX combined with radiation therapy effectively promotes tumor cell apoptosis.

### mtDNA quantity and quality

The mtDNA analysis revealed that DOX treatment may induce compensatory mitochondrial replication following cellular damage, leading to an increase in mitochondrial number (Fig. 3E). However, X-ray irradiation exacerbated mitochondrial damage, resulting in a relative decrease in mtDNA content in the DOX + X-ray group. In contrast, the TPP-DOX treatment group exhibited significant impairment of mitochondrial function. Although subsequent X-ray irradiation may trigger compensatory mitochondrial replication in response to cellular damage, overall mitochondrial function remained compromised, indicating a more severe and sustained mitochondrial dysfunction.

### ROS measurements

To investigate the ROS generation ability of TPP-DOX and DOX, as well as their combined radiation therapy in 4T1 cells, the fluorescent probe DCFH-DA was utilized (Fig. 3F,G). Both TPP-DOX and DOX treatment significantly enhanced the ROS generation of tumor cells compared to the control group. TPP-DOX exhibited slightly higher ROS generation than DOX. After combined treatment with X-ray radiation therapy, the ROS increase in the DOX combined with the X-ray group was not significant, while the mitochondrial targeting drug TPP-DOX combined with X-ray induced a significant increase in cellular ROS levels. This finding further confirms the synergistic ability of TPP-DOX in targeting mitochondria in conjunction with radiotherapy.

### TEM of mitochondria

TME of mitochondria revealed that, compared to the regular and dense mitochondrial structure in the CONTROL group, the TPP-DOX treatment caused significant alterations in 4T1 cells (Fig. 3H). Specifically, the mitochondrial cristae became incomplete and the shape of the mitochondria was rounded. Furthermore, following combined X-ray radiotherapy, there was an increase in intracellular vacuoles, and both the inner and outer mitochondrial membranes appeared blurred. These observations indicate that DOX treatment alone causes less mitochondrial damage, whereas TPP-DOX induces more severe mitochondrial dysfunction. The damage to mitochondria is further exacerbated by radiotherapy.

### Cell scratch

The impact of TPP-DOX on tumor migration was assessed through scratch experiments. Following TPP-DOX treatment, it exhibited a hindering effect on scratch closure when compared to DOX in both the irradiation and non-irradiation groups (Fig. S8).

### Anti-tumor activities in tumor-bearing grafted nude mice

**Figure 4:**
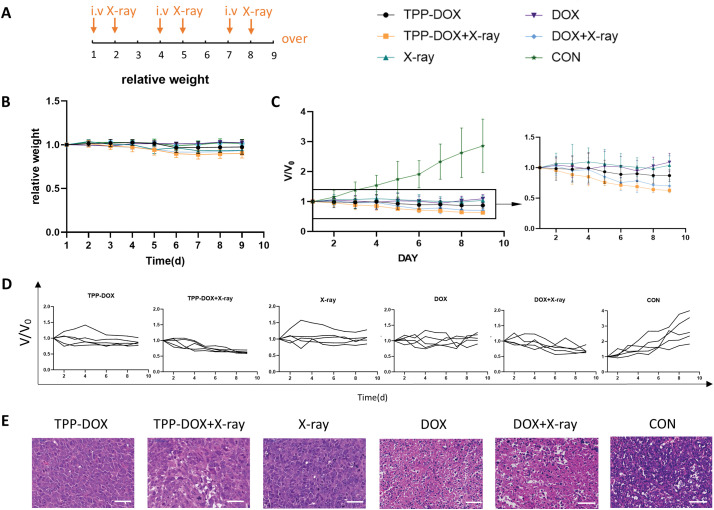
Anti-tumor study of different treatment groups in tumor-bearing grafted nude mice. (A) The time of injection and radiotherapy; (B) the daily body weight change curve of mice during the treatment period (n = 6); (C) the curve of the average relative tumor volume of each group over time, V represents the measured volume of tumor, while V₀ indicates the initial measured volume of tumor before the treatment was applied (n = 6); (D) tumor change curves in each treatment group (n = 6); (E) H&E staining evaluation of tumor tissue (bar = 50 μm).

The weight changes of tumor-bearing grafted nude mice were monitored after treatment with TPP-DOX, DOX, and radiation therapy (Fig. 4A,B). The body weight of the mice in each group remained similar to that before treatment. However, a slight reduction in body weight was observed in the TPP-DOX or DOX combined with radiotherapy and radiotherapy alone groups, possibly due to reduced food intake resulting from radiotherapy.

The tumor size of the treated tumor-bearing grafted nude mice was measured daily, and the tumor volume was calculated. The tumor volume change curve of each treatment group was plotted using the ratio of the tumor volume of each mouse to its initial volume on the first day (Fig. 4C). All treatment groups exhibited significant inhibitory effects on tumor growth compared to the control group. Intratumoral injection of DOX and TPP-DOX alone, as well as radiotherapy alone, effectively inhibited tumor growth. However, the combined treatment group showed a more pronounced tumor-suppressive effect, with TPP-DOX demonstrating good anti-tumor activity. Furthermore, the combination of TPP-DOX with radiation therapy exhibited a synergistic effect (*p* < 0.01). The tumor inhibition curves of individual mice in each group supported these results (Fig. 4D). Additionally, H&E staining of tumor tissues revealed a further reduction in the number of “blue” cell nuclei and evident DNA damage, such as nuclear shrinkage, in the TPP-DOX+X-ray group (Fig. 4E). These findings demonstrate that TPP-DOX combined with radiotherapy enhances the killing effect on tumor cells and confirms the synergistic effect of TPP-DOX with radiotherapy.

**Figure 5:**
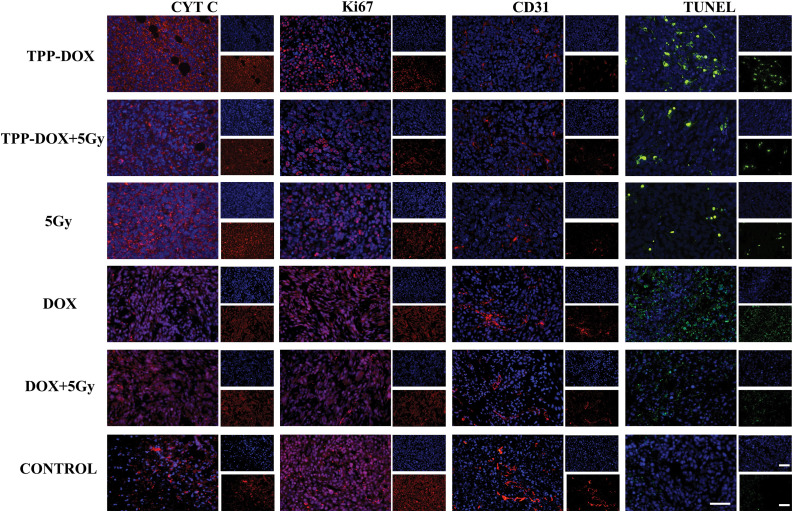
CYT C, Ki67, CD31, TUNEL staining image of mouse tumor tissue after treatment (bar = 50 μm).

To investigate the anti-tumor activity of TPP-DOX, tumor tissues from treated tumor-bearing grafted nude mice were collected and stained with CYT C, Ki67, TUNEL, and CD31 (Fig. 5). The semi-quantitative analysis of the staining images (Fig. S9) of mouse tumor tissue following treatment was conducted to assess the relative expression levels and distribution patterns of key markers.

The staining results in Fig. 5 reveal that the administration of TPP-DOX or radiotherapy leads to the release of a larger amount of CYT C from tumor tissue compared to the control group. This substantial leakage of CYT C indicates mitochondrial damage. The previous findings have established that TPP-DOX enhances the effectiveness of radiotherapy by inducing apoptosis in cancer cells. Therefore, it can be inferred that CYT C might be one of the key components that initiate mitochondria-mediated apoptosis.

Ki67 is a protein associated with tumor proliferation, expressed during various phases (G1, S, G2, M) of the cell cycle but not in the quiescent phase (G0). The detection of the Ki67 index directly reflects the impact of drug treatment on tumor cell proliferation. The results in Fig. 5 demonstrate a significant reduction in the Ki67 protein content in tumor tissue after treatment, indicating the effective inhibition of tumor cell proliferation by TPP-DOX. Moreover, the combination of TPP-DOX with low-dose radiation therapy exhibits a more pronounced reduction in Ki67 protein expression, indicating a synergistic anti-tumor effect.

CD31, a membrane glycoprotein and a member of the immunoglobulin superfamily, plays a role in eliminating aging neutrophils *in vivo*. In immunohistochemical assays, CD31 is used to assess the rate of tumor angiogenesis and growth. In Fig. 5, the protein content of CD31 in tumor tissues of each treatment group is lower compared to the control group. Notably, the combined treatment of TPP-DOX with low-dose radiation therapy results in even lower CD31 protein content, indicating that TPP-DOX exhibits a similar inhibitory effect on tumor growth as DOX, while the combined radiation therapy enhances this effect further.

The TUNEL method, also known as *in situ* end-labeling of DNA breaks, employs FITC-conjugated dUTP and TdT enzymes to detect DNA damage in cells. This method is widely used to study the effects of different drug groups on DNA damage in tumor cells. Fig. 5 demonstrates that TPP-DOX effectively induces tumor DNA damage.

### Organ toxicity evaluation in tumor-bearing grafted nude mice

**Figure 6:**
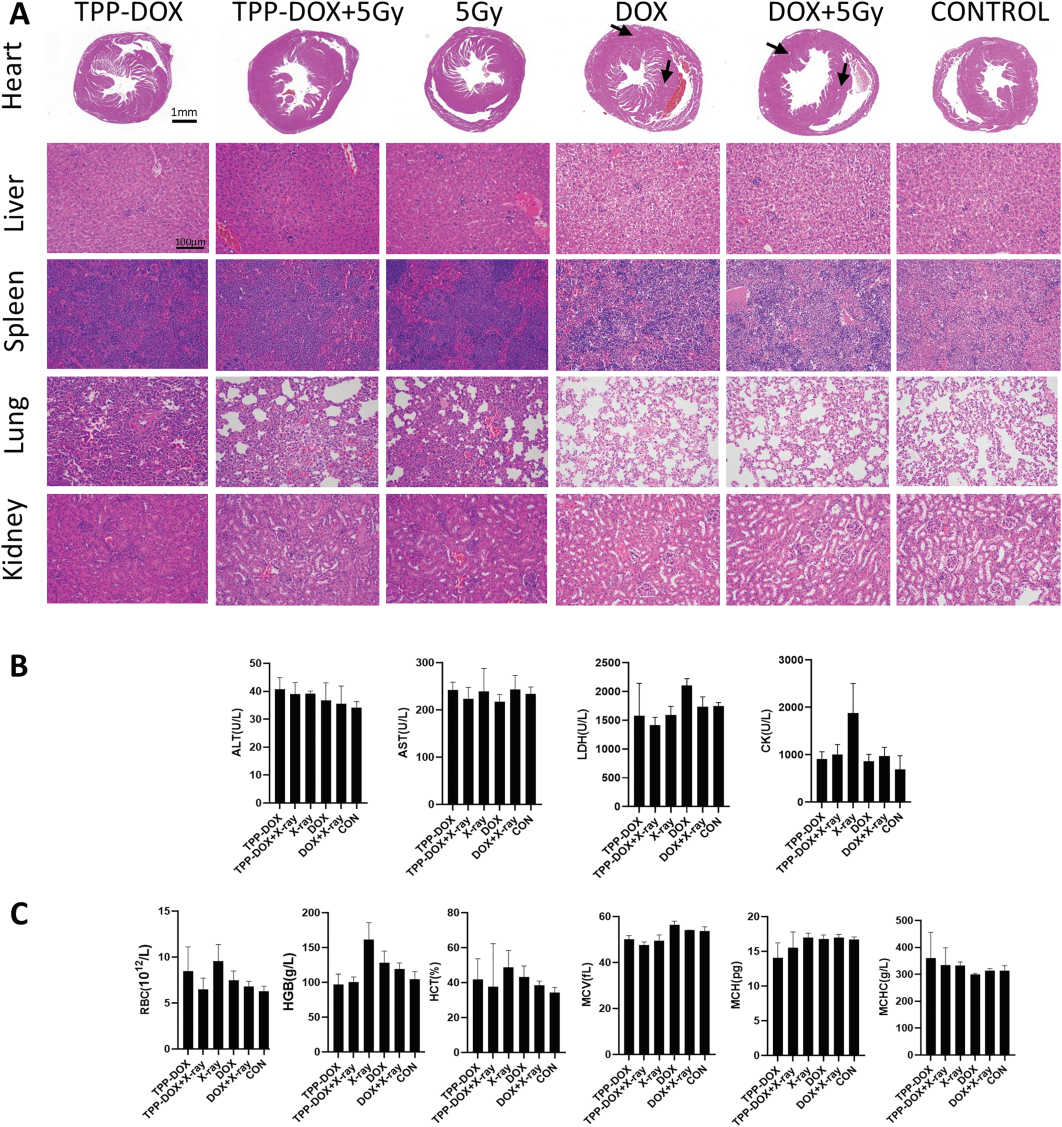
*In vivo* biosafety evaluation of tumor-bearing grafted nude mice in different treatment groups. (A) H&E staining to evaluate organ toxicity. The arrow points to the necrotic tissue; (B) blood biochemical evaluation of liver function and myocardial function in blood samples of mice after treatment (n = 3). ALT (Alanine transaminase), AST (Aspartate transaminase), LDH (Lactate dehydrogenase), CK (Creatine kinase); (C) blood routine evaluation of blood samples of mice after treatment (n = 3). RBS (Red blood cell), HGB (Hemoglobin), HCT (Hematocrit), MCV (Mean corpuscular volume), MCH (Mean corpuscular hemoglobin), MCHC (Mean corpuscular hemoglobin concentration).

H&E staining of major organs, including the heart, liver, kidney, spleen, and lungs, was performed to evaluate organ toxicity in tumor-bearing grafted nude mice after 9 days of treatment (Fig. 6A).

Following intratumoral administration of doxorubicin, the cardiac space in the mice expanded, the muscle fiber texture became irregular, the cytoplasm of cardiomyocytes displayed vacuolation, and cardiomyocyte necrosis occurred.

### Blood routine and blood biochemistry of tumor-bearing grafted nude mice

To assess the safety of the drug *in vivo*, biochemical indicators (Fig. 6B) and blood routine (Fig. 6C) of tumor-bearing grafted nude mice were examined after 9 days of treatment.

There was no significant difference between the group, among which there was a slight increase in lactate dehydrogenase (LDH) in the DOX-treated group. It was considered to be caused by doxorubicin damage to the heart. Compared with the control group, the main blood biochemical and routine blood indexes did not change significantly before and after TPP-DOX combined with radiotherapy, which preliminarily showed that TPP-DOX had better safety and good biocompatibility with radiotherapy.

## Discussion

The structure of TPP-DOX was confirmed, demonstrate the successful development of this mitochondria-targeted drug. The positive charge of the lipophilic cationic TPP enhances TPP-DOX uptake through interaction with negatively charged cell membranes, leading to increased fluorescence co-localization. While free DOX exhibits more pronounced cytotoxicity due to its nuclear binding in sensitive cells, TPP-DOX, with its larger particle size, cannot enter the nucleus, resulting in lower observable toxicity. Our studies indicate that in drug-resistant cells, limited nuclear entry of DOX hampers its effects [56–58], while TPP-DOX promotes apoptosis through mitochondrial targeting, indicating comparable efficacy to DOX in sensitive cells.

TPP-DOX significantly improves uptake in tumor cells, particularly in mitochondria, leading to elevated ROS levels and reduced mitochondrial membrane potential. Moreover, TPP-DOX combined with radiotherapy enhances apoptosis. Literature supports that covalent conjugation of TPP to compounds effectively targets mitochondria, inducing apoptosis in tumor cells. TPP-DOX not only inhibits tumor cell proliferation but also exhibits synergistic effects with low-dose radiotherapy, enhancing anti-tumor activity. Importantly, the TPP-DOX treatment group showed no significant organ damage, indicating its potential to reduce the cardiotoxicity associated with traditional DOX.

Initially, the utilization of liposomes as carriers for encapsulating TPP-DOX was considered to enhance its tumor-targeting efficacy. Given the limited water solubility of TPP-DOX and concerns regarding the systemic side effects typically associated with conventional liposomes, we opted to explore direct intratumoral injection of TPP-DOX in our experimental design. This decision was driven by a dual aim: prioritizing therapeutic efficacy while mitigating potential off-target effects. Nonetheless, for the seamless translation of this approach into clinical settings, further optimization of drug carrier encapsulation is imperative. Furthermore, combinational therapies, such as the inclusion of transcription factor targeting (e.g., Transcription Factor 3), have been explored to enhance therapeutic efficacy and improve treatment outcomes [59]. In this regard, exploring polymer-based methodologies holds promise, as they can leverage physical binding mechanisms to augment TPP-DOX delivery, thus potentially enhancing therapeutic outcomes and addressing the intrinsic challenges associated with targeted drug delivery to tumor. Furthermore, this study demonstrates that TPP-DOX can produce a synergistic therapeutic effect when combined with radiotherapy. However, the combination of chemotherapy and radiotherapy may introduce some complexity in clinical application. Moving forward, research could focus on investigating the synergistic effects of mitochondrial-targeted radiosensitizers, such as TPP-conjugated gold nanoparticles, in direct combination with X-ray radiotherapy. This approach could streamline the treatment regimen, with a primary focus on tumor radiotherapy, making it potentially more convenient for both research and clinical translation.

## Conclusion

The study focused on the synthesis and characterization of TPP-DOX, a mitochondria-targeting drug obtained by modifying DOX with TPP. The study explored the potential of TPP-DOX in combination with radiotherapy for tumor treatment. Exploration of intracellular localization to mitochondria, impact on mitochondrial membrane permeability and ROS levels also outcome to exposure. *In vitro* experiments demonstrated the enhanced cellular uptake, apoptosis induction, and ROS generation by TPP-DOX, indicating its efficacy in targeting mitochondria. *In vivo* studies using a tumor-bearing mouse model further confirmed the antitumor activity and safety of TPP-DOX. In drug-resistant cells, DOX struggles to enter the nucleus, limiting its impact to the cytoplasm [56]. TPP-DOX, entering mitochondria, enhances apoptosis [57], proving effective in both sensitive and drug-resistant tumor cells [56–58]. The findings highlight the promising application prospects of mitochondria-targeted drug delivery combined with complementary therapeutic approaches in the field of anticancer treatment.

## Supplementary Materials

Figure S1 Characterization of TPP-DOX. 1H nuclear magnetic resonance (1H–NMR) spectra of TPP-DOX (A), DOX (B) and TPP (C).

Figure S2 Characterization of TPP–DOX. Mass spectrometry (MS) spectra of TPP–DOX (A), DOX (B) and TPP (C).

Figure S3 Fourier transform infrared (FT–IR) spectral detection of TPP–DOX and DOX. The arrows indicate the characteristic peaks of the substance.

Figure S4 Cytotoxicity results of normal cells (HUVEC and MCF10). A. MCF 10A cytotoxicity investigation of different drug treatment groups (DOX, TPP–DOX); B: MCF 10A cytotoxicity of different drug treatment groups (DOX, TPP–DOX) combined with radiotherapy; C: HUVEC cytotoxicity investigation of different drug treatment groups (DOX, TPP–DOX); D: HUVEC cytotoxicity of different drug treatment groups (DOX, TPP-DOX) combined with radiotherapy.

Figure S5 Fluorescence image of 4T1 cells stained with Calcein AM/PI. The green for live cells and red for dead cells (bar = 200 U+03BC;m).

Figure S6 The graph depicts the concentration of pNA (p–nitroaniline) produced by intracellular Caspase–3 and Caspase–9 catalyzed substrates after treatment of 4T1 cells with DOX or TPP–DOX (n = 3).

Figure S7 ATP concentration and RLU standard curve.

Figure S8 Cell scratch. A Microscopic images of 4T1 cells treated with DOX, TPP–DOX, and combined with X–ray for 24 h and 48 h (bar = 200 U+03BC;m). B The quantitative analysis of Panel A (n=3, *p<0.01, ****p<0.0001).

Figure S9 Semi-quantitative analysis of CYT C (A), Ki67 (B), CD31 (C), TUNEL(D) staining image of mouse tumor tissue after treatment. (n=3, *p<0.05, **p<0.01, ***pπ.001, ****p<0.0001).

## Data Availability

All data generated or analyzed during this study are included in this published article (and its supplementary information files).

## Supplementary Materials

The supplementary material is available online at https://doi.org/10.32604/or.2025.058997.
