# Supplementary figures and images for: Comparative assessment of antitumor effects between doxorubicin and mitochondria-targeted doxorubicin in combination with radiotherapy
Source: Oncol Res. 2025 May 29;33(6):1423–36. doi: 10.32604/or.2025.058997 (PMC12144633; doi:10.32604/or.2025.058997)

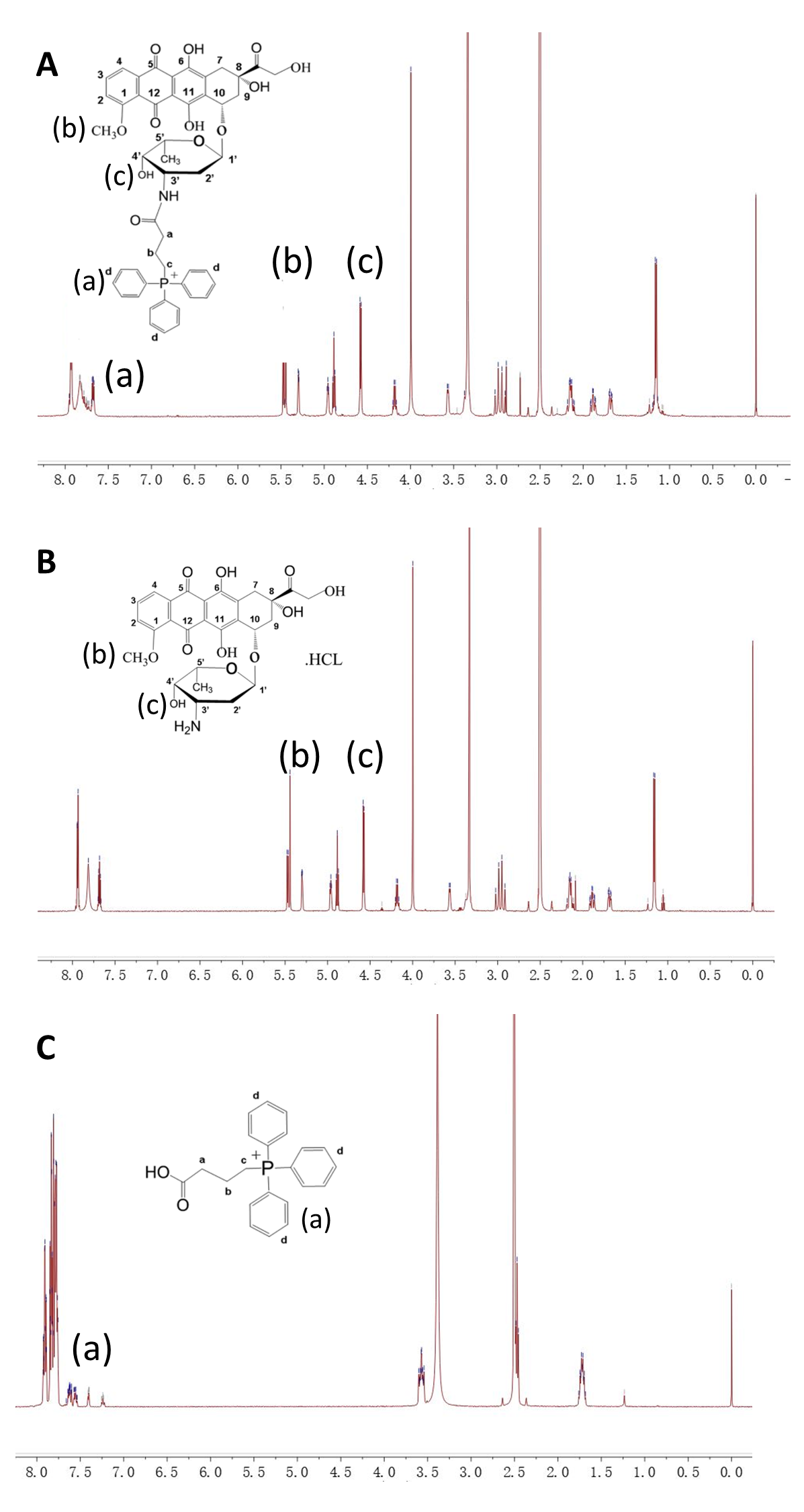

Supplement: Figure S1 [file OncolRes-33-58997-s001.TIF]

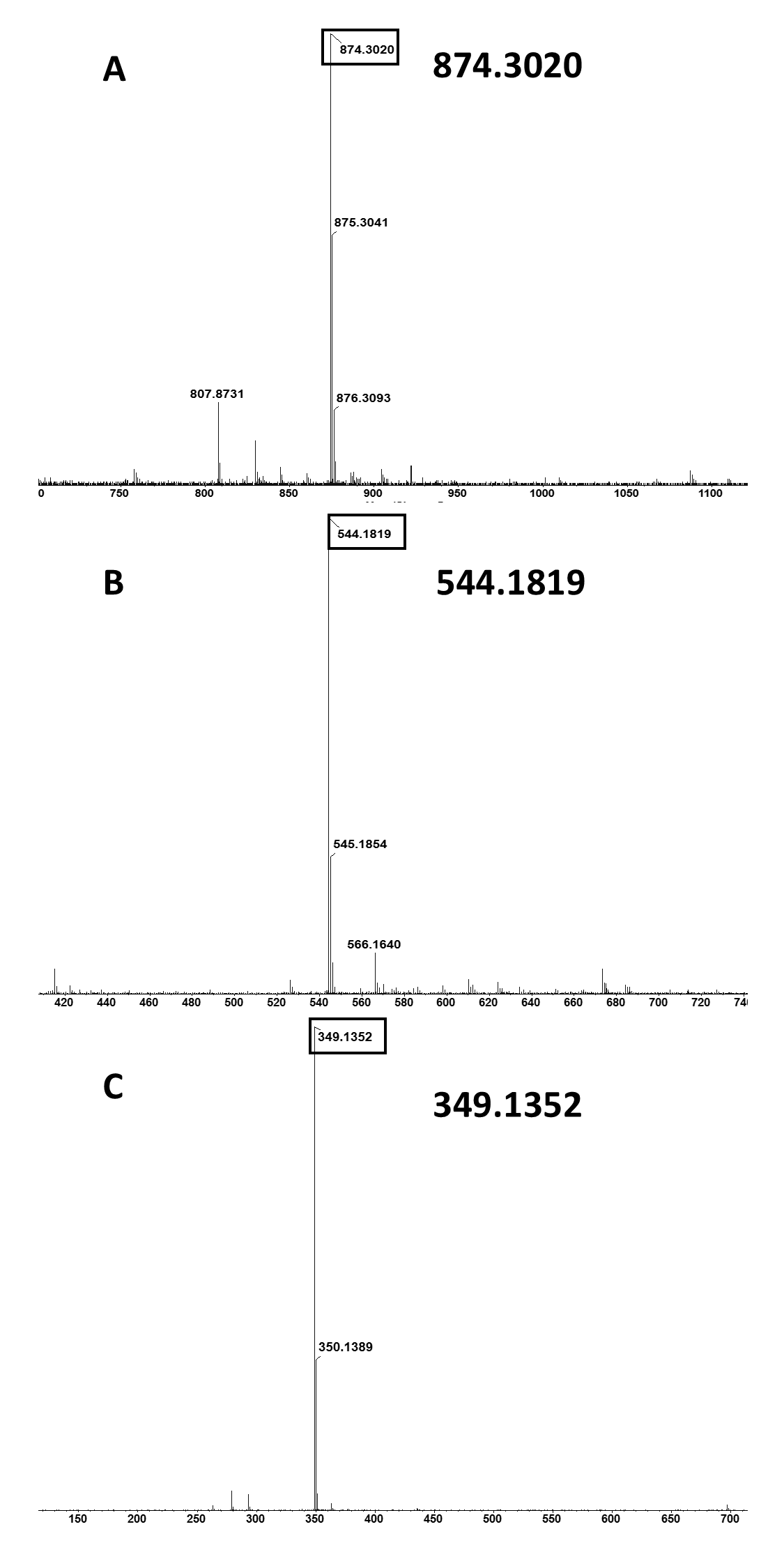

Supplement: Figure S2 [file OncolRes-33-58997-s002.TIF]

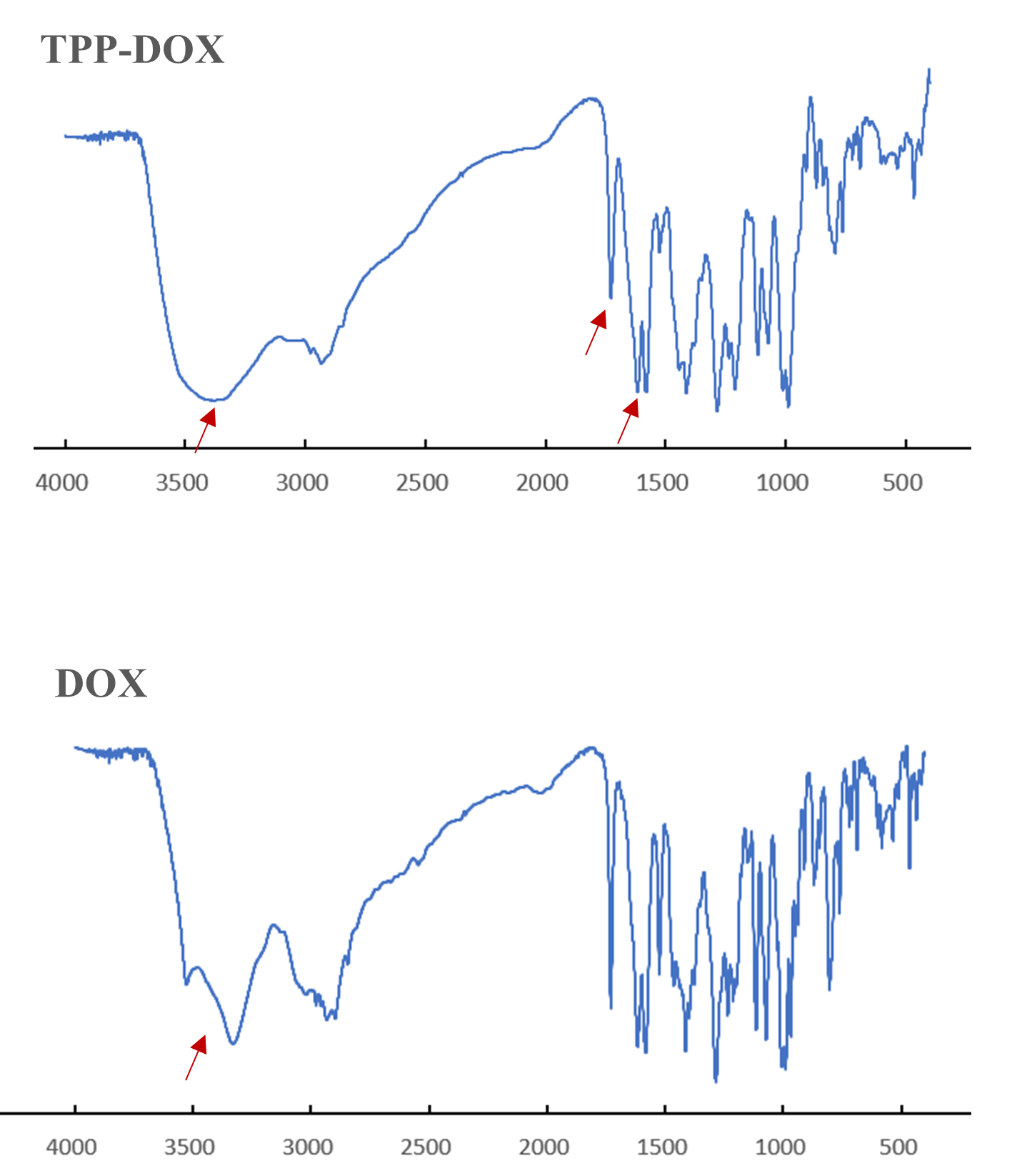

Supplement: Figure S3 [file OncolRes-33-58997-s003.TIF]

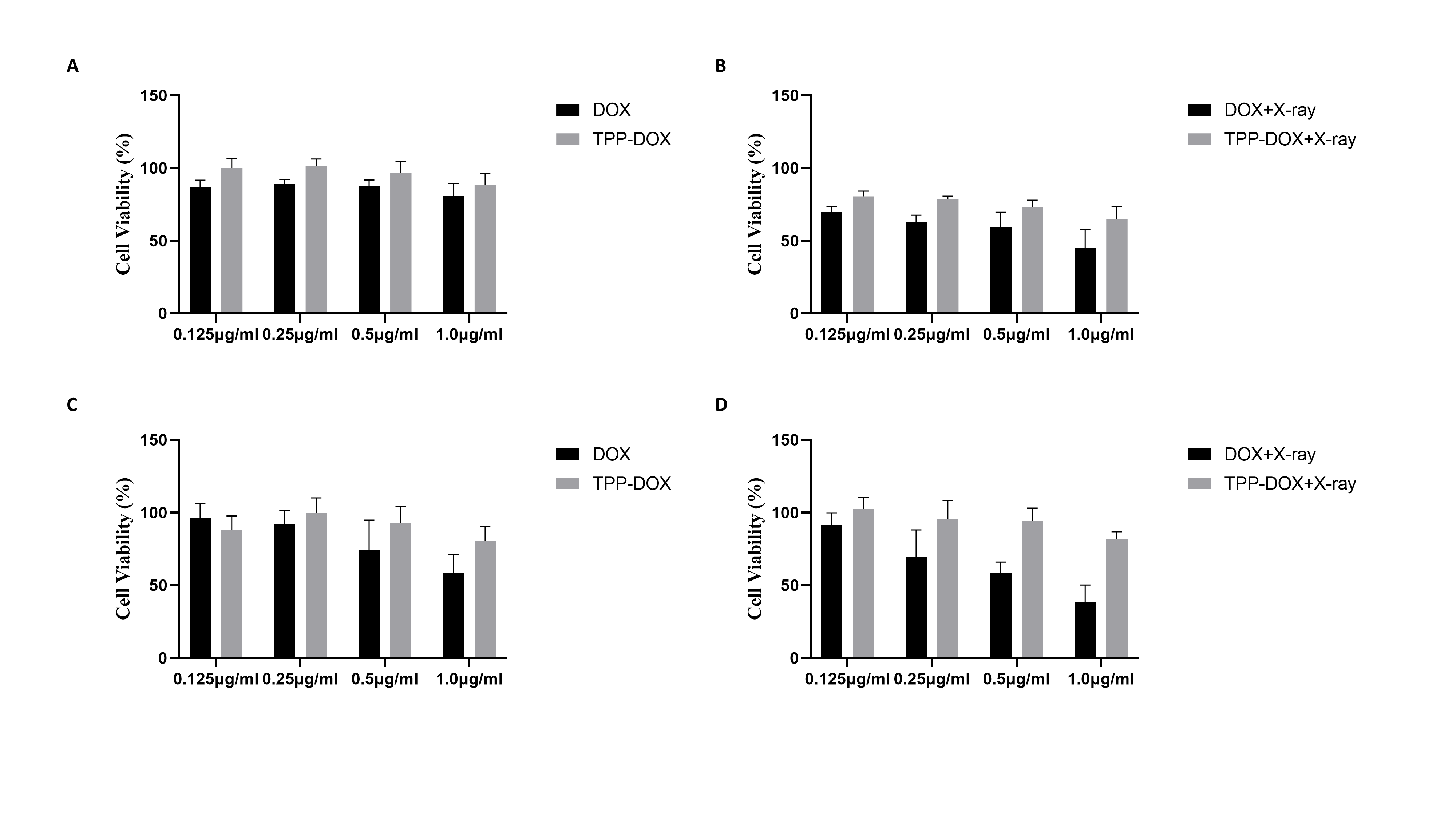

Supplement: Figure S4 [file OncolRes-33-58997-s004.TIF]

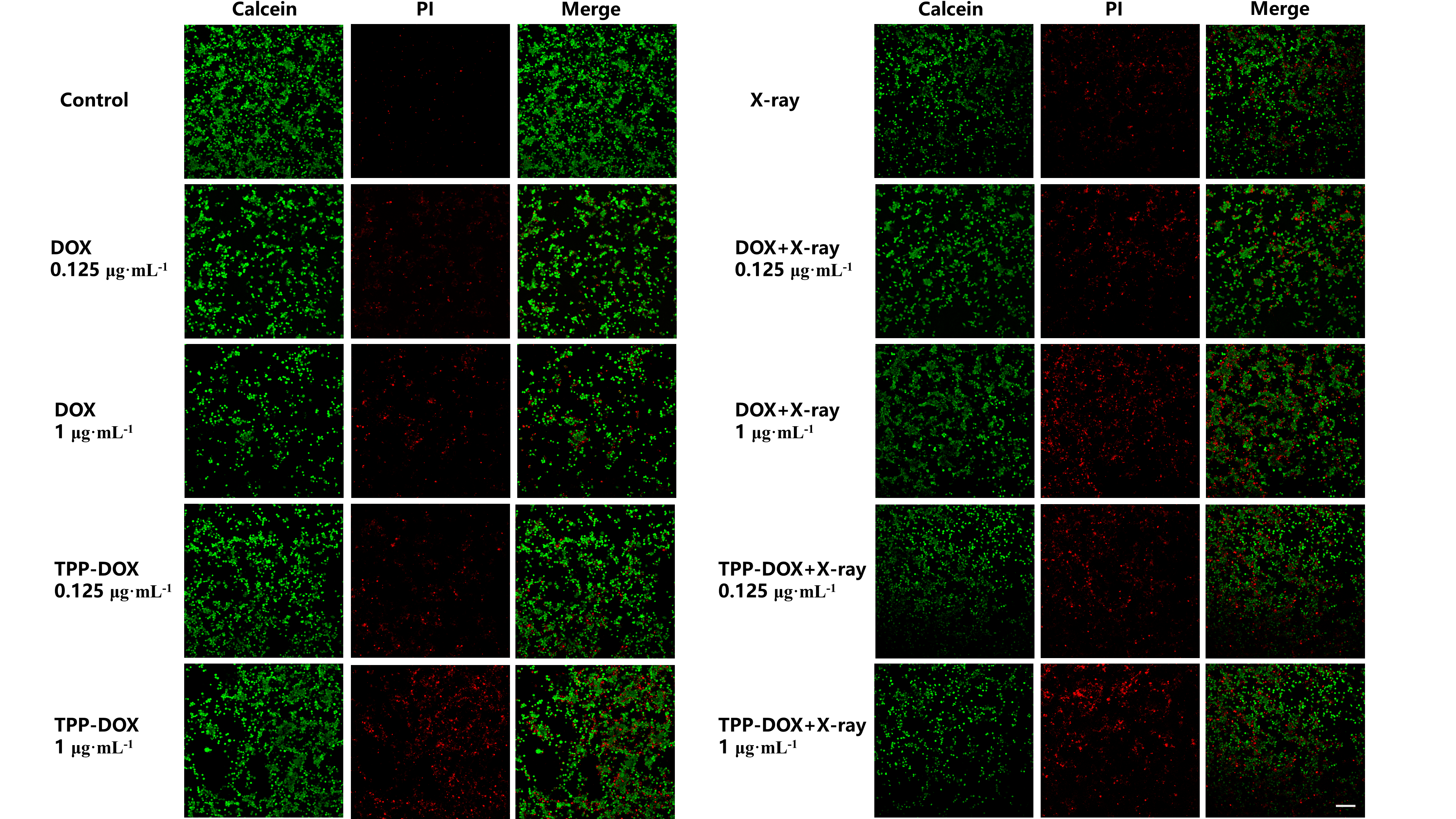

Supplement: Figure S5 [file OncolRes-33-58997-s005.TIF]

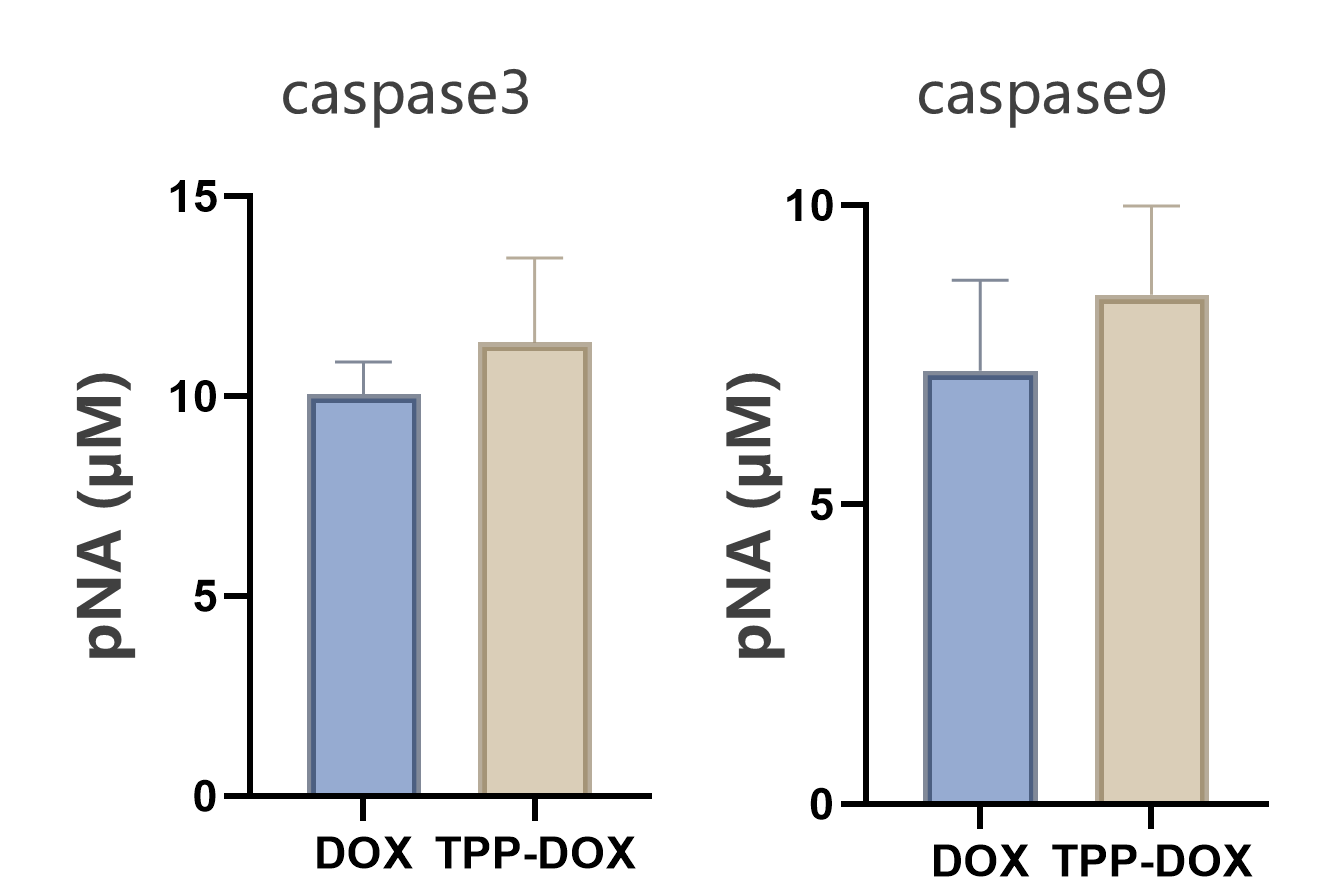

Supplement: Figure S6 [file OncolRes-33-58997-s006.TIF]

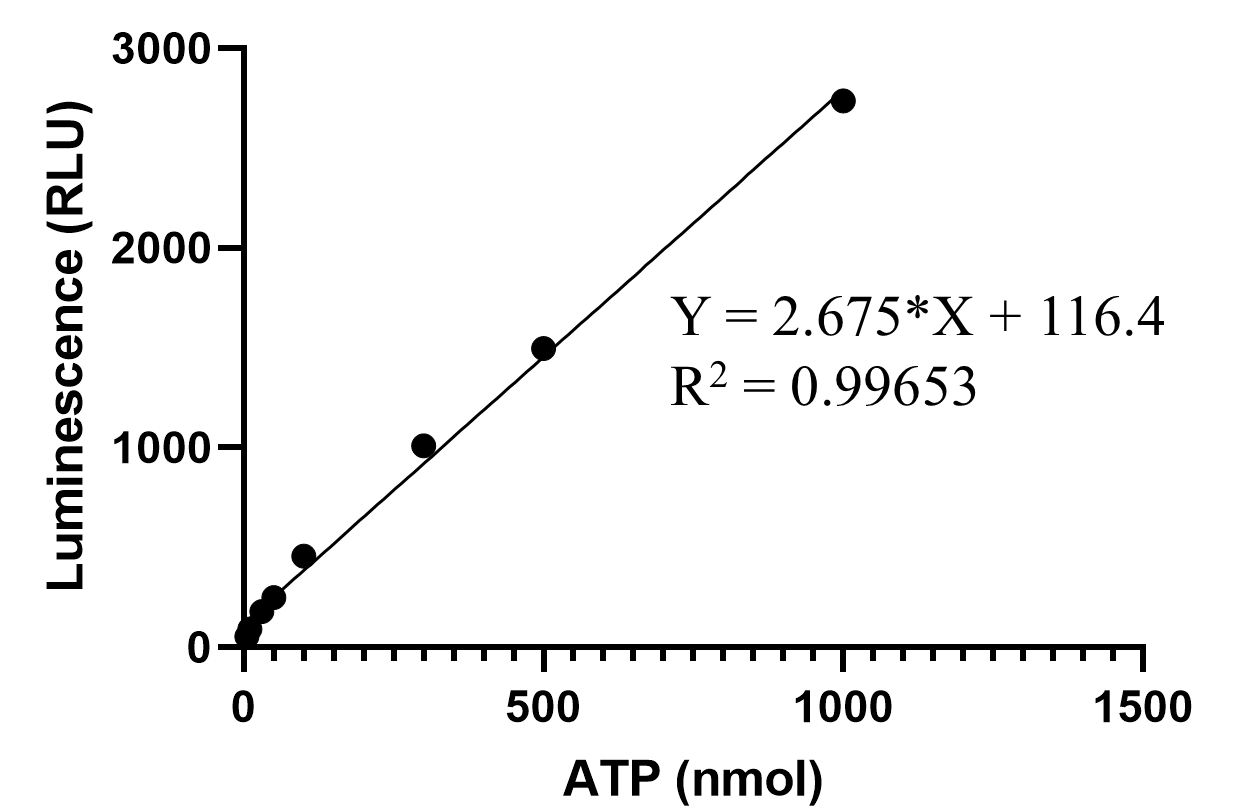

Supplement: Figure S7 [file OncolRes-33-58997-s007.TIF]

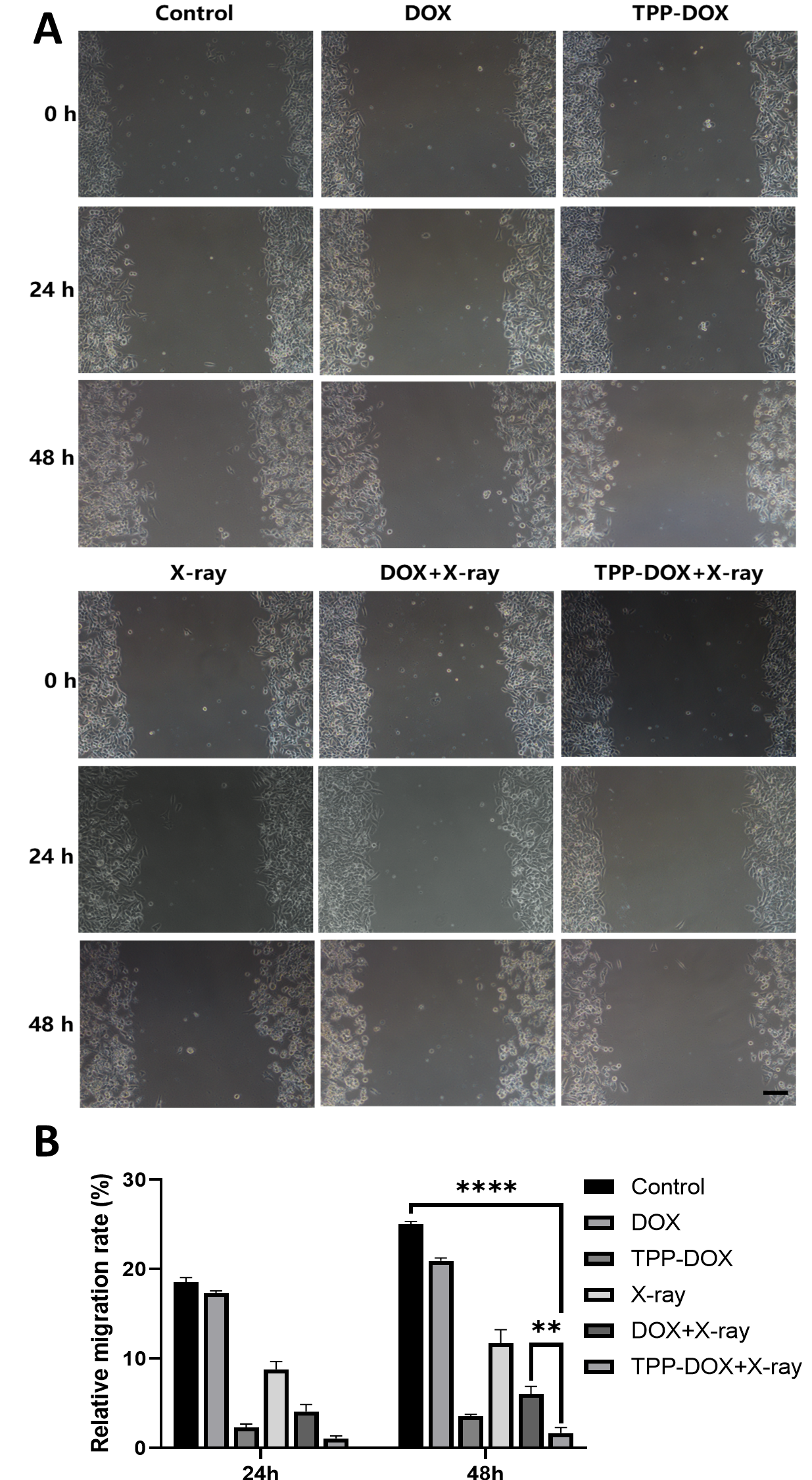

Supplement: Figure S8 [file OncolRes-33-58997-s008.TIF]

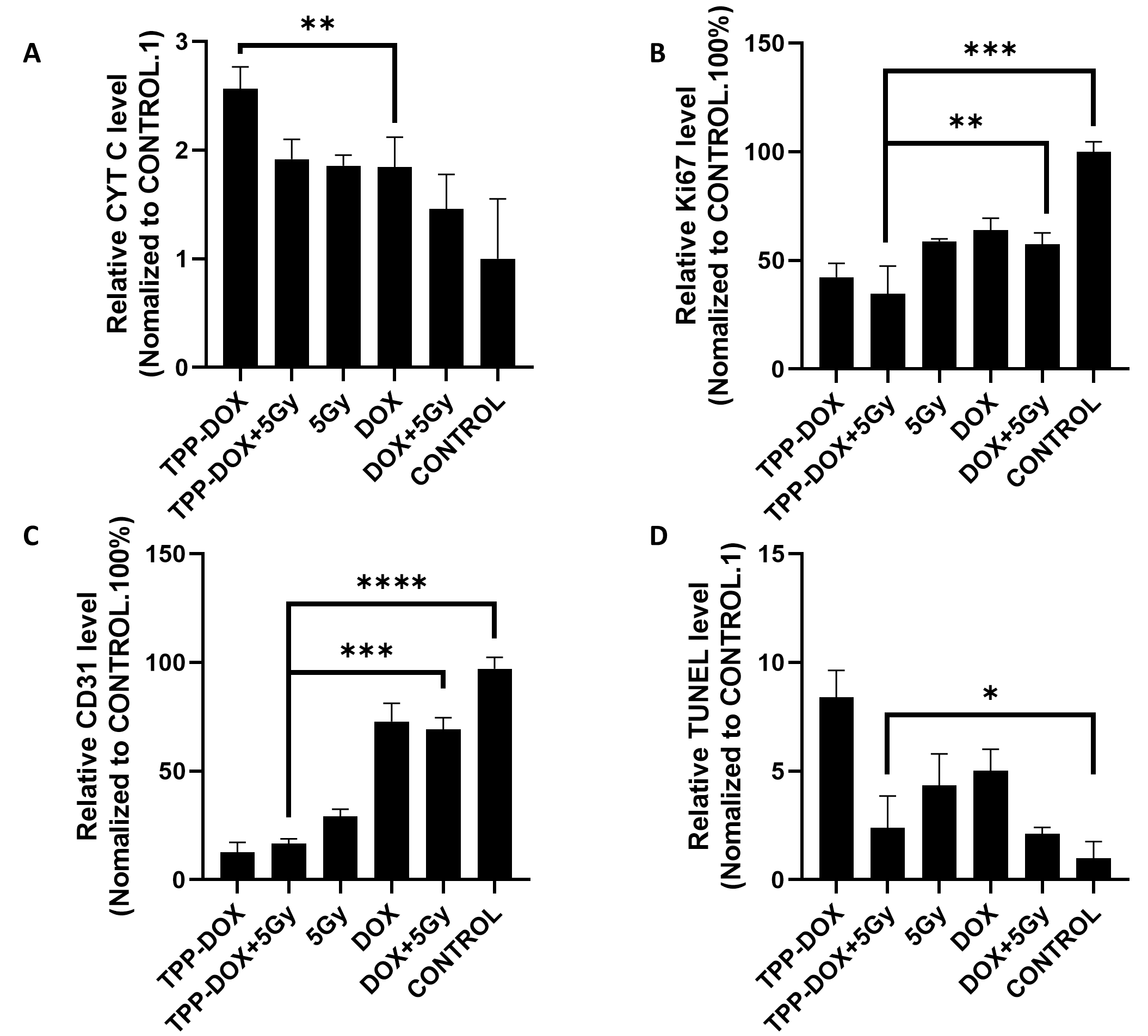

Supplement: Figure S9 [file OncolRes-33-58997-s009.TIF]
